# Effect of suturing in root coverage via coronally advanced flaps: A systematic review

**DOI:** 10.1002/cap.10312

**Published:** 2024-09-14

**Authors:** Alina Ariceta, Leandro Chambrone, Sandra Stuhr, Emilio Couso‐Queiruga

**Affiliations:** ^1^ Department of Periodontology School of Dentistry Catholic University of Uruguay Montevideo Uruguay; ^2^ Evidence‐Based Hub, Centro de Investigação Interdisciplinar Egas Moniz (CiiEM) Egas Moniz School of Health & Science Monte de Caparica Caparica Portugal; ^3^ Unit of Basic Oral Investigations (UIBO) School of Dentistry Universidad El Bosque Bogotá Colombia; ^4^ Department of Periodontics and Oral Medicine University of Michigan School of Dentistry Ann Arbor Michigan USA; ^5^ Department of Oral Surgery and Stomatology University of Bern School of Dental Medicine Bern Switzerland

**Keywords:** esthetics, gingival recession, surgery, surgical flaps

## Abstract

**Background:**

To analyze the evidence about the influence of the suturing technique and material in terms of the percentage of mean root coverage (%MRC) following root coverage therapy in teeth diagnosed with single/localized gingival recession defects (GRD) via a monolaminar coronally advanced flap.

**Methods:**

The protocol of this systematic review was registered in PROSPERO (CRD42024514043). A literature search was conducted to identify investigations that fulfilled the eligibility criteria. Variables of interest were extracted, subsequently categorized, and qualitatively analyzed.

**Results:**

A total of 15 randomized clinical trials, including 301 localized GRD in non‐molar sites classified as Miller class I‐II/RT1, in 253 patients were included. The studies reporting the combination of sling and single interrupted sutures, or interrupted sutures alone showed an MRC of 70.2%±16.6%, and 74.1%±0.75%, respectively. The highest MRC was observed in the studies using polyglactin 910 with a pooled value of 76.6% ± 15.3%, and monofilament materials, with a pooled MRC of 74.8%±7.1%. When the suturing diameter was evaluated, the highest pooled MRC with values of 79.1%±9.8% was observed with the use of 5‐0.

**Conclusions:**

For the treatment of single/localized GRD in non‐molar sites via a monolaminar coronally advanced flap, the use of a combination of sling and single interrupted sutures, or single interrupted sutures, polyglactin 910 or monofilament materials, and material diameter of 5‐0 showed a higher MRC as compared to the use of expanded polytetrafluoroethylene, and silk with/without dressing, and other suture diameters.

**Key points:**

- There were no differences in the percentage of root coverage achieved between the use of sling and single interrupted, versus single interrupted sutures alone on the treatment of single/localized GRD in non‐molar sites.
- Polyglactin 910 and monofilament sutures resulted in a higher percentage of root coverage achieved as compared to expanded polytetrafluoroethylene, and silk with/without dressing.
- The use of 5‐0 material diameter showed the highest percentage of root coverage achieved.

**Plain language summary:**

This study was primarily aimed at evaluating how different suturing techniques and materials could affect the percentage of root coverage in single/localized recession defects, without the use of soft tissue substitutes or autogenous soft tissue grafts. After the pooled analyses of 15 randomized clinical trials that fulfilled the inclusion criteria, we observed that the adequate selection of suturing techniques, materials, and size could lead to a higher percentage of root coverage. Specifically, the use of single interrupted with or without sling sutures at the most coronal portion, Polyglactin 910 or monofilament materials, and size of 5‐0 showed a higher percentage of root coverage as compared to the use of expanded polytetrafluoroethylene, and silk with/without dressing, and other suture diameters.

## INTRODUCTION

Gingival recession defect (GRD) can be defined as an apical migration of the gingival margin respective to the cemento‐enamel junction.^1^ A recent systematic review (SR) including observational studies reported that more than two‐thirds of the population worldwide is affected by this condition.^2^ This partial exposure of the root surface could impact the patient's esthetics, function, and health. If left untreated, there is a significantly increased odds of GRD progression over time.^3^

Successful management of GRDs depends on identifying and controlling the etiological factor(s) that lead to their onset and progression, assessing the phenotypic characteristics of the site,^4^ and selecting the most suitable treatment option.^5^ Root coverage procedures with the aim of repositioning the gingival margin as coronal as possible or to its original position, have shown predictable outcomes for the treatment of single and multiple GRDs.^6^, ^7^, ^8^, ^9^ However, the success of this intervention in terms of mean root coverage (MRC), complete root coverage (CRC), and long‐term stability of treatment outcomes, could be influenced by several factors. These factors include interproximal clinical attachment levels (CAL), tooth position, gingival thickness, amount of keratinized tissue width (KTW), vestibular depth, surgical technique, timing of suture removal, extent of the apical migration of the gingival margin, or use of monolaminar or bilaminar techniques, among others.^7^, ^10^, ^11^, ^12^, ^13^, ^14^, ^15^, ^16^

According to a recent SR on this topic, the most effective therapy for the treatment of GRD in terms of MRC and CRC is a bilaminar technique utilizing a subepithelial connective tissue graft with a coronally advanced flap (CAF), followed by EMD + CAF, acellular dermal matrix + CAF, platelet‐rich fibrin + CAF, xenograft collagen matrix + CAF, and CAF alone. Similarly, MRC and CRC revealed different outcomes in the included investigations.^8^ These findings were also reported in another study, where researchers observed that tooth location (i.e., incisors vs. molars) plays an important role in the achieved outcomes. The variance in the observed findings among studies could be explained by the baseline characteristics of the GRD, which may have influenced the patients’ treatment.^17^

To date, to the best of the authors’ knowledge, no SR has previously analyzed the influence of the suturing technique and material following RC procedures in single/localized GRDs via a monolaminar CAF.^18^ Thus, the primary aim of this SR was to evaluate the influence of the suturing technique and material used in teeth that underwent mucogingival procedures with the aim of root coverage via a monolaminar CAF in terms of percentage of MRC (%MRC). The secondary outcomes were to evaluate the effect of material diameter on the %MRC, and the incidence and type of complications of this type of intervention. Therefore, the following focused question was addressed: “What is the influence of the suturing technique and material on the %MRC, following the treatment of single GRDs with no interproximal clinical attachment loss with the aim of root coverage via a CAF?”

## MATERIALS AND METHODS

The protocol of this review fully adhered to the guidelines of the Preferred Reporting Items of Systematic Reviews and Meta‐Analyses statement,^19^ and was registered in the International Prospective Register of Systematic Reviews (PROSPERO) with the identification code CRD42024514043.

### PICOS outline

“What is the influence of the suturing technique and material on the %MRC, following the treatment of single GRDs with no interproximal clinical attachment loss with the aim of root coverage via a CAF?”

- Population: Adult human subjects (≥18 years of age) in the presence of single/localized GRD categorized as RT1,^10^ or Miller class I and II^13^ that required root coverage procedure.
- Intervention: Root coverage procedure via a monolaminar CAF.
- Comparison: Any possible comparison between suture techniques and materials.
- Outcomes:

Primary outcome:
%MRC.
Secondary outcomes:
The effect of material diameter on %MRC, incidence, and type of complications.

- Study design: Randomized clinical trials (RCTs).

### Eligibility criteria

For inclusion, studies must have been published in English and recruited adult human subjects (≥18 years) requiring the treatment of non‐molar or molar single GRD with no interproximal attachment loss via a monolaminar technique. The intervention of interest was a CAF alone, with no addition of autogenous soft tissue grafts, soft tissue substitute materials, or biologics. The minimum follow‐up period required was ≥6 months. Preclinical studies, non‐randomized controlled trials (non‐RCTs), RCT involving the treatment of multiple recession defects, the use of bilaminar techniques, and RCT investigations not reporting both the type of suturing technique and material used as well as the %MRC were excluded.

### Search methods

Three electronic databases were searched, the National Library of Medicine (MEDLINE – PubMed), Cochrane Central Register of Controlled Trials (CENTRAL), and EMBASE using a specific strategy. The last electronic search was conducted on March 23, 2024. Comprehensive search strategies were designed for each database, based on the use of MeSH terms, keywords, and free terms structured, see Table S1 in Clinical Advances in Periodontics. Additionally, to complement the database search, recently published systematic reviews on this topic were performed by screening the bibliographies of the identified articles.^7^, ^8^, ^14^

### Article selection process

A preliminary selection was performed by two independent reviewers (Alina Ariceta and Emilio Couso‐Queiruga), after reading the title and abstract of entries obtained from the literature searches. Both reviewers read the full‐text versions of potentially eligible studies. Final article selection based on the aforementioned eligibility criteria was performed. When disagreement regarding the final selection of an article occurred, both reviewers had an open discussion. In the case that no agreement was achieved, a third reviewer (Sandra Stuhr) made the final decision. Following article selection, Cohen's kappa coefficient (k) was calculated to determine the degree of inter‐examiner agreement.

### Data extraction

Two authors (Alina Ariceta and Emilio Couso‐Queiruga) performed separately the data extraction and were previously calibrated to ensure consistency in the data extraction process and the terminology employed. A third reviewer (Sandra Stuhr) checked the data accuracy. Any missing data were requested from the corresponding authors of the original articles via email communication. If no response was received within 3 weeks, the requested dataset and/or the investigation was excluded from the final analysis. In addition to the outcomes of interest previously outlined, the following data were extracted and recorded in duplicate by the two independent reviewers: 1. Year of publication and citation; 2. Type of setting, location, and study design; 3. Final number of participants/recession and distribution by groups; 4. Age and sex distribution; 5. Classification, location, characteristics of the site, and total healing period; 6. Patient‐related factors; 7. Type of suturing material and diameter, type of suturing technique, type of root coverage technique; 8. Percentage of root coverage, incidence, and type of complications.

### Risk of bias assessment

The risk of bias analyses of the included investigations were independently performed by two examiners (Alina Ariceta and Sandra Stuhr) using the Cochrane Collaboration tool for assessing the risk of bias in RCTs.^20^ RCTs were categorized as low, with some concerns or high risk of bias. Disagreement between reviewers was resolved by open discussion. In case no agreement could be achieved, the final decision was made by a third reviewer (Emilio Couso‐Queiruga).

### Data synthesis

The extracted data, including study characteristics and results, from the included studies were summarized into qualitative data forms. The descriptive analysis was divided into the following categories: general study characteristics, risk of bias, and relevant endpoints.

## RESULTS

### Search results and study selection

**FIGURE 1:**
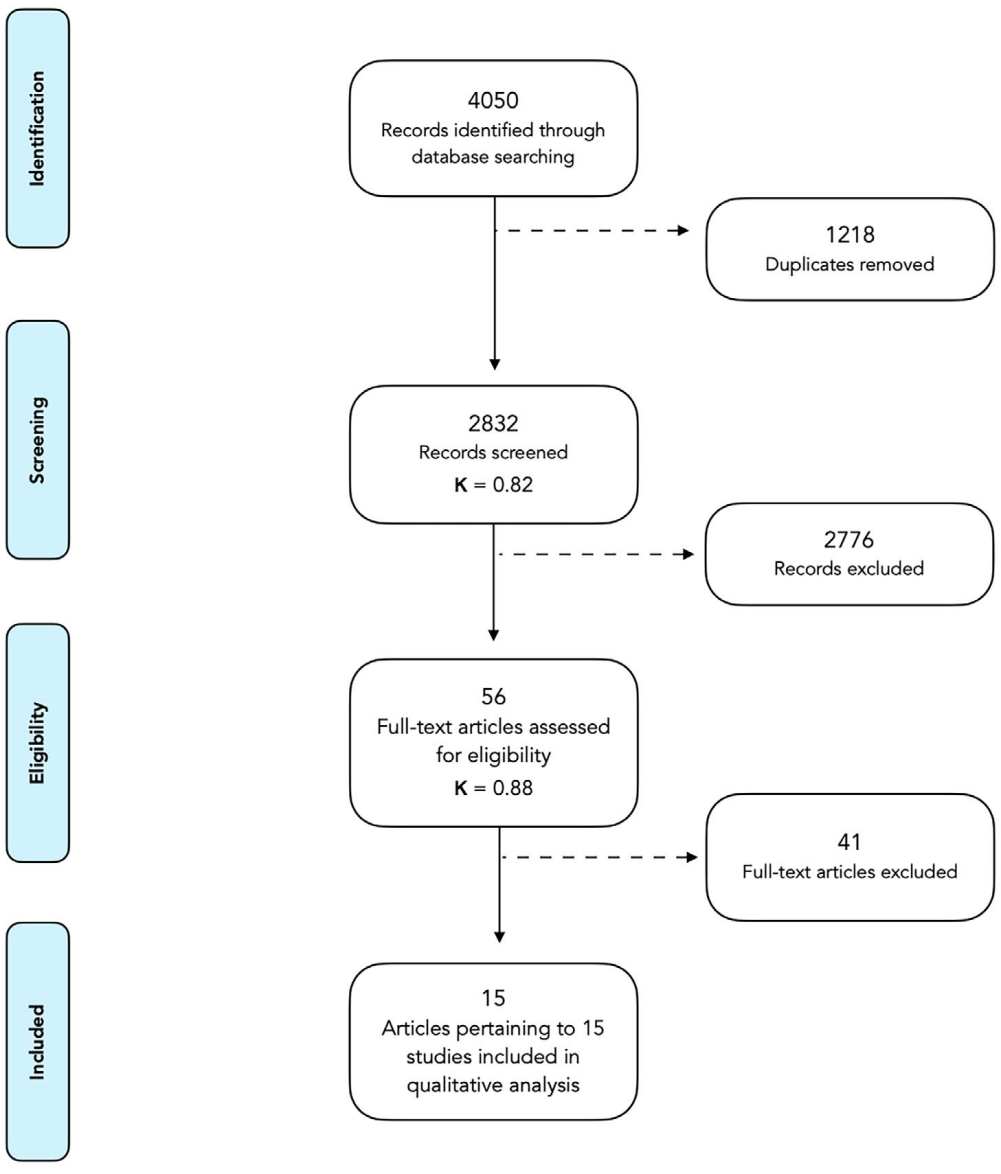
Preferred Reporting Items for Systematic Reviews and Meta‐Analyses (PRISMA) flowchart.

The initial database search yielded a total of 4050 records, of which 1498 were found in Pubmed, 1427 in EMBASE, and 1125 in CENTRAL. Following duplicate removal, 2832 entries remained. After title and abstract screening, a total of 56 articles were selected for full‐text review. Forty‐one articles did not meet the inclusion criteria. See Table S2 in online *Clinical Advances in Periodontics*. Therefore, the final selection was comprised of 15 RCTs.^21^, ^22^, ^23^, ^24^, ^25^, ^26^, ^27^, ^28^, ^29^, ^30^, ^31^, ^32^, ^33^, ^34^, ^35^ Quantitative analysis could not be performed due to the high methodological heterogeneity among studies. Thus, a thorough descriptive analysis of the reported outcomes was performed. Kappa scores for inter‐examiner agreement for title and abstract review as well as full‐text review were 0.82 and 0.88, respectively. The article selection process is depicted in Figure 1.

### Study characteristics

All studies were conducted in a university setting,^21^, ^22^, ^23^, ^24^, ^25^, ^26^, ^27^, ^28^, ^30^, ^31^, ^33^, ^34^, ^35^ except two multicenter studies that did not specify where the study was performed.^29^, ^32^ The final sample consisted of approximately 253 patients since one study did not specify the number of patients in the control group,^23^ with a total of 301 localized non‐molar GRDs (premolar to premolar) classified as RT1 or Miller class I or II. All included studies reported the treatment of single GRDs via a monolaminar CAF.

Twelve studies utilized sling sutures at the most coronal part and single interrupted sutures at the vertical incisions.^21^, ^23^, ^24^, ^26^, ^27^, ^28^, ^29^, ^31^, ^32^, ^33^, ^35^ Contrarily, three studies reported the use of single interrupted sutures only.^22^, ^25^, ^34^ Four studies utilized silk as material,^21^, ^23^, ^25^, ^33^ of which three of them also used periodontal dressing.^23^, ^25^, ^33^ One study reported the use of expanded polytetrafluoroethylene (ePTFE),^32^ and seven studies used polyglactin 910.^22^, ^24^, ^26^, ^27^, ^30^, ^31^, ^34^ Finally, three studies used monofilament alone (i.e., nylon).^28^, ^29^, ^35^ The diameter of the suture material varied from 3‐0 to 6‐0 in the included studies, except for the study by Moreira et al. which did not specify it.^26^

The type of root coverage procedure consisted of split‐full‐split,^21^, ^22^, ^23^, ^24^, ^29^, ^31^, ^34^ full‐split,^25^, ^26^, ^27^, ^28^, ^30^, ^33^, ^35^ and partial‐full thickness approaches.^32^ The mean follow‐up was 11.6 ± 14.5 months, ranging between 6 and 60 months. The general characteristics of the included studies, mucogingival procedure, study subjects, and site features are displayed in Tables 1 and 2.

**TABLE 1:** General characteristics, study subjects, and site features of the included studies.

General Study Information					Study Subjects and Site Features													
Publication(s) Year and Author(s)	Setting	Location (Country)	Study Design Parallel arms or Split‐mouth	Final Number of Participants/recession and Distribution	Age Distribution (Years)	Sex Distribution	Single Defects	Classification (RT1, Miller I, Miller II)	Location Non‐Molar / Molar teeth	Maxillary / Mandibular	Gingival Recession Depth	KTW	Gingival Thickness	Probing depth	Clinical Attachment Level	Healing Time / Period	Time of suture removal	Patient‐related Factors (i.e., smoking, diabetes, history of periodontitis)
2023 Santamaria et al.	University	Pescara, Italy	Parallel	20 participants / 20 recessions	18 ‐ 47	NR	Yes	RT1	Non‐molar	Maxillary	4.00 ± 0.65	1.65 ± 0.59	1.07 ± 0.44	0.95 ± 0.22	4.95 ± 0.69	6 months	14 days	Systemically healthy, no smokers
2017 Sangiorgio et al.	University	Piracicaba and São Paulo, Brazil	Parallel	17 participants / 17 recessions	38.12 ± 12.95	6 M/11F	Yes	Miller Class I and II	Non‐molar	Maxillary	3.22 ± 0.45	2.86 ± 1.30	0.94 ± 0.30	1.50 ± 0.75	4.72 ± 0.86	6 months	15 days	Systemically healthy
2017 Kumar et al.	University	Lucknow, India	Parallel	N/R participants / 15 recessions	35.53 ± 6.52	NR	Yes	Miller Class I and II	Non‐molar	Maxillary	2.00 ± 0.53	4.13 ± 1.30	1.66 ± 0.23	2.00 ± 0.84	4.00 ± 0.65	6 months	10 days	Systemically healthy
2017 Jepsen et al.	University	Germany, and Italy	Split‐mouth	16 participants / 16 recessions	44 ± 15 years (20 ‐ 73 years)	8 M/10F	Yes	Miller Class I or II	Non‐molar	Maxillary / Mandibular	3.11 ± 0.78	2.22 ± 1.39	0.96 ± 0.34	1.50 ± 0.54	4.61 ± 0.87	3 years	Vertical sutures: 7 days Sling suture: 14 days	No taking medications and smokers< 10 cigarettes/day
2016 Shivakumar et al.	University	Tumkur, India	Split‐mouth	10 participants / 10 recessions	28.41 ± 7.15 (18 ‐ 45)	8 M/2F	Yes	Miller Class I or II	NR	NR	3.30 ± 0.95	1.90 ± 0.57	1.13 ± 0.08	2.60 ± 0.52	5.90 ± 1.29	6 months	10 days	Systemically healthy
2016 Moreira et al.	University	Sao Paolo, Brazil	Parallel	14 participants / 20 recessions	34.7 ± 9.63	6 M/14F	Yes	Miller Class I or II	Non‐molar	Maxillary	3.14 ± 0.51	2.60 ± 0.94	1.01 ± 0.18	1.15 ± 0.37	4.24 ± 0.64	6 months	14 days	Nonsmokers and systemically healthy
2014 Kumar et al.	University	Virajpet, India	Split‐mouth	10 participants / 10 recessions	NR	7 M/3F	Yes	Miller Class I	Non‐molar	Maxillary	2.9 ± 0.73	NR	NR	2.0 ± 0.47	NR	6 months	7 days	Systemically healthy
2013 Kuis et al.	University	Rijeka, Croatia	Split‐mouth	37 participants / 57 recessions	31.14 (20 ‐ 52)	12 M/25F	Yes	Miller Class I or II	NR	NR	2.63 ± 0.75	1.33 + 1.19	NR	2.65 + 0.73 (1‐4)	3.79 + 0.77	5 years	14 days	Nonsmokers and systemically healthy
2013 Jepsen et al.	NR	Germany, Italy, Sweden, Spain	Split‐mouth	45 participants / 45 recessions	39.5 ± 13.8 (20 ‐ 73)	28F/17M	Yes	Miller Class I or II	Non‐molar	Maxillary	3.34 ± 1.00	2.00 ± 1.22	0.89 ± 0.34	1.48 ± 0.65	4.82 ± 1.09	6 months	Vertical sutures: 7 days Sling suture: 14 days.	Systemically healthy and smokers <10 cigarettes/day
2010 Jagannathachary and Prakash	University	Karnataka, India	Split‐mouth	10 participants / 10 recessions	37 (20‐55)	5F/5M	Yes	Miller Class II	Non‐molar	Maxillary	2.20 ± 0.95	3.75 ± 0.79	1.00 ± 0.24	2.40 ± 0.52	4.60 ± 0.81	6 months	14 days	Systemically healthy
2009 Zucchelli et al.	University	Bologna, Italy	Split‐mouth	11 participants (CAF + curets; CAF + ultrasonic) / 22 recessions	31.6 (18‐40)	4 M/7F	Yes	Miller Class I	Non‐molar	Maxillary	(CAF + curets) 3.64 ± 0.80; (CAF + ultrasound) 3.82 ± 0.60	(CAF + curets) 1.63 ± 0.67; (CAF + ultrasound) 1.72 ± 0.64	NR	(CAF + curets) 1.09 ± 0.09; (CAF + ultrasound) 1.09 ± 0.30	(CAF + curets) 4.72 ± 0.78; (CAF + ultrasound) 4.90 ± 0.54	6 months	14 days	Systemically healthy and smokers ≤10 cigarettes/day
2009 Cortellini et al.	NR	Italy	Parallel	43 participants / 43 recessions	37.8 ± 8.4 (25−59)	23 M/20F	Yes	Miller Class I or II	Non‐molar	Maxillary	2.4 ± 0.7	3.2 ± 1.3	NR	1.2 ± 0.4	3.7 ± 0.8	6 months	7‐9 days	9 smokers
2007 Mahajan et al.	University	Lucknow, India	Parallel	14 participants / 14 recessions	25.2 (16‐40)	7 M/7F	Yes	Miller Class I or II	Non‐molar	Maxillary / Mandibular	3.71 ± 0.75	3.42 ± 4.14	NR	1.85 ± 0.60	NR	6 months	7 days	NR
2005 Del Pizzo et al.	University	Turin and Bologna, Italy	Split‐mouth	15 participants / 15 recessions	39.46 ± 10.72 (18‐56)	4 M/11F	Yes	Miller Class I and II	Non‐molar	Maxillary	4.13 ± 0.74	1.67 ± 0.82	NR	1 ± 0	5.13 ± 0.74	2 years	14 days	Systemically healthy
2004 Da Silva et al.	University	Piracicaba, Brazil	Split‐mouth	11 participants / 11 recessions	29.2 (18‐43)	6 M/5F	Yes	Miller Class I	Non‐molar	Maxillary	3.98 ± 0.62	3.38 ± 1.53	1.27 ± 0.29	1.47 ± 0.45	5.45 ± 0.76	6 months	14 days	Systemically healthy

**TABLE 2:** Mucogingival procedure, and relevant endpoints of the included studies.

	Mucogingival Procedure(s)				Relevant Endpoints	
Publication(s) Year and Author(s)	Type of Suturing Material	Diameter of Suturing Material	Type of Suture Technique (i.e., single or sling)	Type of Root Coverage Technique (i.e., Split‐Full‐Split, Tunneling)	Percentage of Root Coverage (Mean ± SD)	Number and Type of Complications
2023 Santamaria et al.	Silk	4‐0	Sling and simple (in vertical incisions)	Split‐Full‐Split	75%	NR
2017 Sangiorgio et al.	Polyglactin 910	5‐0	Simple	Split‐Full‐Split	68.04 ± 24.11%	NR
2017 Kumar et al.	Silk + Dressing	6‐0	Sling and simple (in vertical incisions)	Split‐Full‐Split	53.3% ± 40.4%	NR
2017 Jepsen et al.	Polyglactin 910	6‐0	Sling and simple (in vertical incisions)	Split‐Full‐Split	82.78% ± 17.04%	NR
2016 Shivakumar et al.	Silk + Dressing	3‐0	Simple	Full‐split	67.52%	NR
2016 Moreira et al.	Polyglactin 910	NR	Sling and simple (in vertical incisions)	Full‐split	72.1% ± 14.4%	NR
2014 Kumar et al.	Polyglactin 910	5‐0	Sling and simple (in vertical incisions)	Partial‐thickness	61.67% ± 30.22%	NR
2013 Kuis et al.	Monofilament	5‐0	Sling and simple (in vertical incisions)	Partial‐thickness	Miller Class I: 83.1% Miller Class II: 82.2% Combined: 82.7 ± 23.8%	NR
2013 Jepsen et al.	Monofilament	6‐0	Sling and simple (in vertical incisions)	Split‐Full‐Split	72.66% ± 26.19%	NR
2010 Jagannathachary and Prakash	Polyglactin 910	4‐0	Sling and simple (in vertical incisions)	Partial‐thickness	53% ± 22.0%	NR
2009 Zucchelli et al.	Polyglactin 910	6‐0	Sling and simple (in vertical incisions)	Split‐Full‐Split	(CAF + curets) 95.4%; (CAF+ ultrasound) 84.2%	NR
2009 Cortellini et al.	Polytetrafluoroethylene	6‐0	Sling and simple (in vertical incisions)	Partial‐Full	37%	NR
2007 Mahajan et al.	Silk + Dressing	4‐0	Sling and simple (in vertical incisions)	Full‐split	77.42%	NR
2005 Del Pizzo et al.	Polyglactin 910	5‐0	Simple	Split‐Full‐Split	86.67% ± 18.29%	NR
2004 Da Silva et al.	Monofilament	6‐0	Sling and simple (in vertical incisions)	Partial‐thickness	69%	NR

### Risk of bias and quality assessment

**FIGURE 2:**
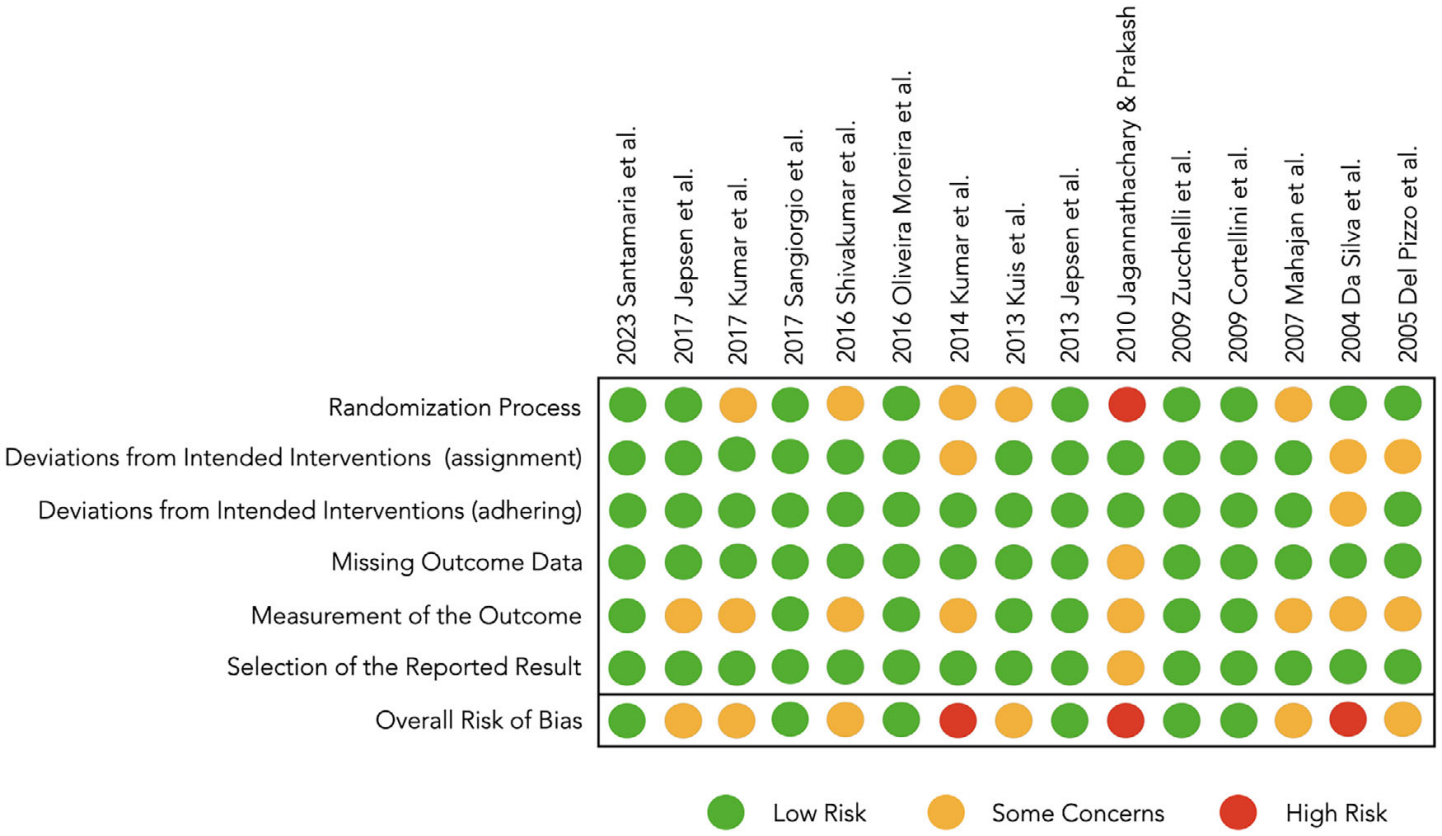
Risk of bias and quality assessment of the randomized controlled trials (RCTs).

Six studies were categorized as presenting low risk,^21^, ^22^, ^26^, ^29^, ^31^, ^32^ six as some concerns,^23^, ^24^, ^25^, ^28^, ^33^, ^34^ and three as high risk of bias^27^, ^30^, ^35^ as displayed in Figure 2.

### Qualitative assessment of outcomes

Data pertaining to the evaluated outcomes of interest are displayed in Tables 1 and 2.

#### Effect of suturing technique on the percentage of root overage

For the studies that utilized a combination of sling and single interrupted sutures,^21^, ^23^, ^24^, ^26^, ^27^, ^28^, ^29^, ^30^, ^31^, ^32^, ^33^, ^35^ the pooled MRC was 70.2% ± 16.6%. In the three studies reporting the use of single interrupted sutures alone, the pooled MRC was 74.1% ± 0.75%.^22^, ^25^, ^34^

#### Effect of suturing material on the percentage of root coverage

In the included studies reporting the use of silk sutures, the MRC varied from 53.3% to 77.4%, with a pooled mean of 68.3% ± 10.9%.^21^, ^23^, ^25^, ^33^ In the studies utilizing silk sutures and dressing, the MRC varied from 53.3% to 77.4%, with a pooled mean of 66.1% ± 12.1%.^23^, ^25^, ^33^ For the only study utilizing ePTFE, the mean MRC was 37%,^32^ whereas the investigations including the use of polyglactin 910 reported a pooled MRC of 76.6% ± 15.3%, ranging from 53% to 95.4%.^22^, ^24^, ^26^, ^27^, ^30^, ^31^, ^34^ Finally, the observed pooled MRC of the studies using monofilament was 74.8% ± 7.1%, ranging from 69.0% to 82.7%.^28^, ^29^, ^35^

#### Effect of suturing diameter on the percentage of root coverage

The only study reporting the use of 3‐0 showed a mean MRC of 67.5%.^25^ In the studies reporting the use of 4‐0, the MRC was 68.5% ± 13.5%, ranging from 53% to 77.4%.^21^, ^30^, ^33^ An MRC of 79.1% ± 9.8%, ranging from 68.04% to 86.7%, was observed in the studies reporting the use of 5‐0.^22^, ^27^, ^28^, ^34^ Finally, the use of 6‐0 was related to an MRC of 65.5% ± 29.5%, ranging from 37% to 95.4%.^23^, ^24^, ^29^, ^31^, ^32^, ^35^

### Incidence and type of complications

None of the studies reported complications during the healing period.

## DISCUSSION

### Summary of findings and quality of the evidence

To the best of our knowledge, this is the first SR evaluating the effect of suturing techniques, materials, and diameters on the %MRC after root coverage therapy in non‐molar teeth diagnosed with single/localized GRD via a monolaminar coronally advanced flap with a minimum follow‐up of 6 months. Data collected in this review indicate that the suturing technique whether it is single interrupted sutures alone, or in combination with a sling suture at the coronal part does not have a significant difference between them in the %MRC achieved. However, sites that were treated with polyglactin 910 and monofilament sutures exhibited a higher %MRC as compared to the use of silk alone, silk with dressing, or ePTFE materials. Additionally, the use of a 5‐0 suture diameter demonstrated the highest %MRC as compared to <5‐0, and 6‐0 material diameters. Interestingly, none of the studies reported any type of complications during the healing period.

### Agreements and disagreements with other studies or reviews

The studies included in this SR only reported the use of single interrupted sutures alone or in combination with a sling suture. The findings from these studies showed almost no differences between suturing techniques in terms of %MRC. Therefore, it seems that single interrupted sutures with/without slings are effective suturing techniques in order to stabilize the monolaminar CAF without excessive flap tension during the initial phases of healing. These results could be explained by the observations by Pini‐Prato and coworkers, who observed that the lower the flap tension, the higher the recession reduction.^36^ Nonetheless, a study by Tavelli and colleagues on cadavers demonstrated differences in the use of slings compared to simple interrupted sutures on the marginal flap stability following CAF, which could impact the outcomes of therapy.^37^ However, due to its natural design, this study evaluated marginal flap stability and not its effect on root coverage.

When the effect of the suturing material was evaluated, a higher %MRC in sites treated with polyglactin 910 and monofilament sutures was observed. These findings could be explained by the dissimilarities in tissue response between materials. As reported by a classic histological study by Lilly and colleagues, differences in mild and severe tissue responses were observed between suture materials. Monofilament sutures were associated with less severe tissue reactions than multifilament materials such as silk.^38^ Similarly, an animal study involving beagle dogs showed that multifilament sutures have a greater conductance of bacteria and inflammatory response than monofilament sutures, with silk causing the most significant inflammatory response.^39^ More recent studies have also demonstrated that suture material could influence the healing process.^40^, ^41^, ^42^ For example, a lower inflammatory reaction was noted with the use of monofilament as compared to multifilament materials.^40^, ^41^, ^42^ Nonetheless, although ePTFE material has been associated with less inflammatory reaction and plaque accumulation,^38^, ^41^ due to the inclusion of only one study utilizing this material in this SR,^32^ no conclusions could be drawn. The best outcomes observed in this study with the use of polyglactin 910 could be also related to the preservation of the tensile strength for a longer period of time as compared to other nonabsorbable sutures^43^ and for the removal of the sutures in a maximum period of approximately 2 weeks. As reported in an SR aimed at evaluating the influence of timing of suture removal in the outcomes of RC therapy, early suture removal (<10 days post‐operative) seems to have a detrimental effect in terms of the percentage of root coverage as compared to sites where the sutures were removed 10 days after surgery.^15^ Comparably, it was also observed that the suture material did not influence the therapeutic outcomes if the removal occurred approximately 10 days after the surgical procedure.^15^

In the clinical decision‐making process, it is crucial for clinicians to consider the advantages and disadvantages of various suture materials to meet the individual needs of the patient and according to the specific clinical scenario.^44^ For instance, while silk sutures offer good knot security, cost‐effectiveness, and easy handling, they also pose the risk of rapid bacterial colonization, thereby increasing the likelihood of inflammation and potential infection.^44^ Conversely, the use of synthetic absorbable sutures such as polyglactin 910 has demonstrated a half‐life tensile strength of 2 weeks, triggering minimal to slight local inflammatory reactions.^44^ Additionally, the use of synthetic nonabsorbable sutures, like nylon, has shown good knot security, easy handling, and minimal inflammatory tissue reaction, while pTFE sutures, due to their high degree of smoothness and memory, have been associated with reduced knot security.^44^ Therefore, careful consideration of these factors is essential in selecting the most appropriate suture material for clinical applications. The characteristics of the suture materials may explain the findings in this study, and their varying biomechanical behavior during the early stages of healing may account for differences in wound stabilization and further therapeutic outcomes.

To the best of our knowledge, no other studies have evaluated the effect of material diameter on the outcome of RC therapy. In this SR we observed that 5‐0 sutures achieved the highest %MRC compared to other material diameters (i.e., <5‐0 and 6‐0). These findings could be explained by the superior tensile strength and knot security achieved by the 5‐0 suture as compared to 6‐0. As reported in an in vitro study, thicker sutures, independently of whether it was nonabsorbable or absorbable, provide superior tensile strength and knot security than thinner sutures, leading to potentially better flap stability during the initial stages of healing, even in the presence of slight flap non‐passivity.^45^ Furthermore, achieving proper flap passivity is an additional surgical factor that could affect clinical outcomes, particularly when using thinner sutures (i.e., 6‐0, and 7‐0). As demonstrated in other studies, if there is adequate flap passivity, its adaptation could be improved by the use of thinner sutures (i.e., 6‐0, and 7‐0) since they will break before the tissues are torn, and could ensure an optimal flap stabilization promoting an adequate early healing process, therefore achieving optimal surgical outcomes.^40^, ^46^

Finally, The results of this study show that the effectiveness of using CAF alone in non‐molar sites is similar to the findings of high‐quality evidence on this topic.^8^, ^14^, ^47^, ^48^ However, it was not possible to compare the effectiveness of this therapy between molar and non‐molar sites since none of the studies included in the analysis examined this treatment in molar areas. According to a study by Zucchelli and colleagues, the location within the posterior and anterior zones, as well as between lower and upper maxillae, may affect the success of this therapy, suggesting that outcomes could be less favorable in posterior sites.^17^

### Limitations in the review process

Although this SR adhered to high methodological standards, it is not exempt from limitations. First, only RCTs including the treatment of non‐molar single/localized GRD via a monolaminar technique utilizing CAF were included. Nevertheless, this could also be considered a strength. The decision was made to homogenize the study sample to avoid the influence of other treatment alternatives or the use of bilaminar techniques. Second, none of the studies included in this review compared different suturing techniques, materials, and diameters, and therefore this type of intervention could not be assessed quantitatively. Third, there was limited information regarding local and systemic factors, regular maintenance protocols, predisposing factors (i.e., tooth brushing, gingival phenotype), lack of the use of other therapies such as biologics, or the skill of treating clinicians (i.e., residents or experienced surgeons) among others that could potentially influence the outcomes of RC therapy. Consequently, these factors could not be evaluated and taken into consideration. Moreover, the reported findings should be interpreted with caution in other clinical scenarios involving multiple GRDs, molar sites, localized and more severe GRDs, the use of other flap designs, autogenous soft tissue grafts harvested from other intraoral locations, and harvesting techniques.^49^, ^50^, ^51^ Hence, future studies on this topic should be conducted utilizing reproducible parameters and methods of assessment and improve the report of information related to local (i.e., dimensions of the gingival phenotype, gingival recession width and length, and gingival thickness), systemic (i.e., diabetes and smoking status), behavioral (frequency of maintenance recalls and tooth brushing technique), and surgical related variables (i.e., suturing technique and material and diameter) on the outcomes of root coverage therapy in mucogingival procedures around teeth.

## CONCLUSIONS

Within the limitations of this SR in the treatment of single GRD in non‐molar sites with no interproximal tissue loss via a monolaminar CAF, it can be concluded that:

1. The combination of sling and single interrupted sutures, or single interrupted sutures showed a similar %MRC.
2. The use of polyglactin 910 and monofilament sutures rendered a higher %MRC as compared to ePTFE, and silk with/without dressing.
3. The use of a 5‐0 material diameter showed the highest %MRC as compared to diameters of 6‐0, or <5‐0.

## AUTHOR CONTRIBUTIONS

Alina Ariceta, Leandro Chambrone, and Emilio Couso‐Queiruga conceived the idea; Alina Ariceta and Emilio Couso‐Queiruga screened the initial entries, selected the articles, and collected the data. Alina Ariceta and Sandra Stuhr assessed the risk of bias. Alina Ariceta, Leandro Chambrone, Sandra Stuhr, and Emilio Couso‐Queiruga, contributed to the design of the final manuscript and analyzed the data. Emilio Couso‐Queiruga led the writing. All the authors critically revised the manuscript.

## CONFLICT OF INTEREST STATEMENT

The authors declare no conflict of interest.

## Supporting information

Supporting Information

Supporting Information

## Data Availability

Data supporting the findings of this study are available upon reasonable request to the corresponding author.
