# Supplementary material for: Effect of suturing in root coverage via coronally advanced flaps: A systematic review
Source: Clin Adv Periodontics. 2024 Sep 14;15(2):179–90. doi: 10.1002/cap.10312 (PMC12266327; doi:10.1002/cap.10312)
Supplement: Supplementary file 1 — Supporting Information [file CAP-15-179-s002.docx]

**APPENDIX**

**Supplementary Table S1.**  The search strategy used in each of the three databases.

| #1 gingival recession |
| --- |
| #2 (recession NEAR gingiva*) OR (recession NEAR defect*) OR ("recession‐type defect*) #3 (exposure NEAR root*) OR (gingiva* NEAR defect*) #4 “gingival augmentation” OR “keratinized tissue augmentation*” #5 #1 OR #2 OR #3 OR #4 #6 "connective tissue graft*" OR "connective‐tissue graft*"  #7 "soft tissue graft*" OR “soft tissue substitute” #8 "coronally advanced flap*"  #9 “dermal matrix graft” OR “acellular dermal matrix graft” OR “allogenous graft” |
| #10 “xenogenous graft” OR “matrix graft” #11 “enamel matrix protein” OR “autologous blood-derived products” OR “platelet-rich plasma” OR “platelet-rich fibrin” OR “platelet rich fibrin” OR “platelet-derived growth factor” #12 #6 OR #7 OR #8 OR #9 OR #10 OR #11 |
| #14 #5 AND #12 |
