# Supplementary material for: Effect of suturing in root coverage via coronally advanced flaps: A systematic review
Source: Clin Adv Periodontics. 2024 Sep 14;15(2):179–90. doi: 10.1002/cap.10312 (PMC12266327; doi:10.1002/cap.10312)
Supplement: Supplementary file 2 — Supporting Information [file CAP-15-179-s001.docx]

**APPENDIX**

**Supplementary Table S2.**  Excluded articles and reasons for exclusion

| **Articles excluded** | **Reason for exclusion** |
| --- | --- |
| 2000 Modica et al.^1^ | Missing suturing technique |
| 2002 Hägewald et al.^2^ | Missing suturing technique |
| 2003 Zucchelli et al.^3^ | Missing treatment of interest |
| 2004 De Queiroz Côrtes et al.^4^ | Missing suturing technique |
| 2005 Burkhardt et al.^5^ | Missing treatment of interest |
| 2005 Huang et al.^6^ | Non-RCT |
| 2005 Zucchelli et al.^7^ | Non-RCT |
| 2006 Bittencourt et al.^8^ | No suture technique used |
| 2006 De Queiroz et al.^9^ | Missing suturing technique |
| 2007 de Sanctis et al.^10^ | Non-RCT |
| 2008 Santamaria et al.^11^ | Missing suturing technique |
| 2009 Bittencourt et al.^12^ | No suture technique used |
| 2009 Santamaria et al.^13^ | Missing suturing technique |
| 2010 Santana et al.^14^ | Missing suturing technique |
| 2011 Pini-prato et al.^15^ | Missing suturing technique and material |
| 2012 Cairo et al.^16^ | Inclusion of RT2 defects |
| 2012 Zucchelli et al.^17^ | Missing treatment of interest |
| 2013 Padma et al.^18^ | Missing suturing material |
| 2014 Moka et al.^19^ | Missing suturing material |
| 2015 Bhandari et al.^20^ | Missing suturing technique and material |
| 2015 Cairo et al.^21^ | Missing suturing material |
| 2015 Gupta et al.^22^ | Missing suturing material |
| 2016 Bellver-Fernandez et al.^23^ | Non-RCT |
| 2016 Stefanini et al.^24^ | Missing suturing material |
| 2016 Zucchelli et al.^25^ | Missing suturing material |
| 2017 Santos et al.^26^ | Missing treatment of interest |
| 2018 Clementini et al.^27^ | Missing suturing material |
| 2018 Dixit et al.^28^ | Missing primary outcome of interest |
| 2018 Francetti et al.^29^ | Non-RCT |
| 2018 Rasperini et al.^30^ | Missing primary outcome of interest |
| 2020 Rasperini et al.^31^ | Non-RCT |
| 2021 Gil et al.^32^ | Missing suturing technique |
| 2022 Evginer et al.^33^ | Missing suturing technique |
| 2022 Garg et al.^34^ | Non-RCT |
| 2022 Lombardo et al.^35^ | Non-RCT |
| 2022 Nath et al.^36^ | Missing primary outcome of interest |
| 2022 Potharaju et al.^37^ | Missing suturing technique |
| 2022 Sharma et al.^38^ | Inclusion of RT2 defects |
| 2023 Cairo et al.^39^ | Inclusion of RT2 defects |
| 2023 Jagtap et al.^40^ | Missing suturing material |
| 2023 Parlak et al.^41^ | Inclusion of RT2 defects |

**REFERENCES**

1. Modica F, Del Pizzo M, Roccuzzo M, Romagnoli R. Coronally advanced flap for the treatment of buccal gingival recessions with and without enamel matrix derivative. A split-mouth study. *Journal of periodontology.* 2000;71(11):1693‐1698.

2. Hägewald S, Spahr A, Rompola E, Haller B, Heijl L, Bernimoulin JP. Comparative study of Emdogain and coronally advanced flap technique in the treatment of human gingival recessions. A prospective controlled clinical study. *Journal of clinical periodontology.* 2002;29(1):35‐41.

3. Zucchelli G, Amore C, Sforza NM, Montebugnoli L, De Sanctis M. Bilaminar techniques for the treatment of recession-type defects. A comparative clinical study. *Journal of clinical periodontology.* 2003;30(10):862‐870.

4. De Queiroz Côrtes A, Martins ÂG, Nociti Jr FH, Sallum AW, Casati MZ, Sallum EA. Coronally positioned flap with or without acellular dermal matrix graft in the treatment of Class I gingival recessions: A randomized controlled clinical study. *Journal of Periodontology.* 2004;75(8):1137-1144.

5. Burkhardt R, Lang NP. Coverage of localized gingival recessions: comparison of micro- and macrosurgical techniques. *Journal of clinical periodontology.* 2005;32(3):287‐293.

6. Huang LH, Neiva RE, Wang HL. Factors affecting the outcomes of coronally advanced flap root coverage procedure. *J Periodontol.* 2005;76(10):1729-1734.

7. Zucchelli G, De Sanctis M. Long-term outcome following treatment of multiple Miller Class I and II recession defects in esthetic areas of the mouth. *Journal of Periodontology.* 2005;76(12):2286-2292.

8. Bittencourt S, Del Peloso Ribeiro E, Sallum EA, Sallum AW, Nociti FH, Casati MZ. Comparative 6-month clinical study of a semilunar coronally positioned flap and subepithelial connective tissue graft for the treatment of gingival recession. *Journal of periodontology.* 2006;77(2):174‐181.

9. de Queiroz C√¥rtes A, Sallum AW, Casati MZ, Nociti FH, Jr., Sallum EA. A two-year prospective study of coronally positioned flap with or without acellular dermal matrix graft. *J Clin Periodontol.* 2006;33(9):683-689.

10. de Sanctis M, Zucchelli G. Coronally advanced flap: a modified surgical approach for isolated recession-type defects: three-year results. *J Clin Periodontol.* 2007;34(3):262-268.

11. Santamaria MP, Suaid FF, Casati MZ, Nociti Jr FH, Sallum AW, Sallum EA. Coronally positioned flap plus resin-modified glass ionomer restoration for the treatment of gingival recession associated with non-carious cervical lesions: A randomized controlled clinical trial. *Journal of Periodontology.* 2008;79(4):621-628.

12. Bittencourt S, Ribeiro Edel P, Sallum EA, Sallum AW, Nociti FH, Casati MZ. Semilunar coronally positioned flap or subepithelial connective tissue graft for the treatment of gingival recession: a 30-month follow-up study. *Journal of periodontology.* 2009;80(7):1076‐1082.

13. Santamaria MP, Da Silva Feitosa D, Nociti Jr FH, Casati MZ, Sallum AW, Sallum EA. Cervical restoration and the amount of soft tissue coverage achieved by coronally advanced flap: A 2-year follow-up randomized-controlled clinical trial. *Journal of Clinical Periodontology.* 2009;36(5):434-441.

14. Santana RB, Mattos CM, Dibart S. A clinical comparison of two flap designs for coronal advancement of the gingival margin: semilunar versus coronally advanced flap. *Journal of clinical periodontology.* 2010;37(7):651‐658.

15. Pini Prato G, Rotundo R, Franceschi D, Cairo F, Cortellini P, Nieri M. Fourteen-year outcomes of coronally advanced flap for root coverage: follow-up from a randomized trial. *Journal of clinical periodontology.* 2011;38(8):715‐720.

16. Cairo F, Cortellini P, Tonetti M, et al. Coronally advanced flap with and without connective tissue graft for the treatment of single maxillary gingival recession with loss of inter-dental attachment. A randomized controlled clinical trial. *Journal of clinical periodontology.* 2012;39(8):760‐768.

17. Zucchelli G, Marzadori M, Mele M, Stefanini M, Montebugnoli L. Root coverage in molar teeth: a comparative controlled randomized clinical trial. *Journal of clinical periodontology.* 2012;39(11):1082‐1088.

18. Padma R, Shilpa A, Kumar PA, Nagasri M, Kumar C, Sreedhar A. A split mouth randomized controlled study to evaluate the adjunctive effect of platelet-rich fibrin to coronally advanced flap in Miller's class-I and II recession defects. *J Indian Soc Periodontol.* 2013;17(5):631-636.

19. Moka LR, Boyapati R, M S, D NS, Swarna C, Putcha M. Comparison of coronally advanced and semilunar coronally repositioned flap for the treatment of gingival recession. *J Clin Diagn Res.* 2014;8(6):Zc04-08.

20. Bhandari R, Uppal RS, Kahlon KS. Comparison of semilunar coronally advanced flap alone and in combination with button technique in the treatment of Miller's Class I and II gingival recessions: a pilot study. *Indian journal of dental research.* 2015;26(6):609‐612.

21. Cairo F, Cortellini P, Tonetti M, et al. Stability of root coverage outcomes at single maxillary gingival recession with loss of interdental attachment: 3-year extension results from a randomized, controlled, clinical trial. *Journal of clinical periodontology.* 2015;42(6):575‐581.

22. Gupta S, Banthia R, Singh P, Banthia P, Raje S, Aggarwal N. Clinical evaluation and comparison of the efficacy of coronally advanced flap alone and in combination with platelet rich fibrin membrane in the treatment of Miller Class I and II gingival recessions. *Contemp Clin Dent.* 2015;6(2):153-160.

23. Bellver-Fernández R, Martínez-Rodriguez AM, Gioia-Palavecino C, Caffesse RG, Peñarrocha M. Surgical treatment of localized gingival recessions using coronally advanced flaps with or without subepithelial connective tissue graft. *Medicina oral, patologia oral y cirugia bucal.* 2016;21(2):e222‐228.

24. Stefanini M, Jepsen K, de Sanctis M, et al. Patient-reported outcomes and aesthetic evaluation of root coverage procedures: a 12-month follow-up of a randomized controlled clinical trial. *Journal of clinical periodontology.* 2016;43(12):1132‐1141.

25. Zucchelli G, Stefanini M, Ganz S, Mazzotti C, Mounssif I, Marzadori M. Coronally Advanced Flap with Different Designs in the Treatment of Gingival Recession: a Comparative Controlled Randomized Clinical Trial. *International journal of periodontics & restorative dentistry.* 2016;36(3):319‐327.

26. Rocha Dos Santos M, Sangiorgio JPM, Neves F, et al. Xenogenous Collagen Matrix and/or Enamel Matrix Derivative for Treatment of Localized Gingival Recessions: A Randomized Clinical Trial. Part II: Patient-Reported Outcomes. *J Periodontol.* 2017;88(12):1319-1328.

27. Clementini M, Discepoli N, Danesi C, de Sanctis M. Biologically guided flap stability: the role of flap thickness including periosteum retention on the performance of the coronally advanced flap-A double-blind randomized clinical trial. *Journal of clinical periodontology.* 2018;45(10):1238‐1246.

28. Dixit N, Lamba AK, Faraz F, Tandon S, Aggarwal K, Ahad A. Root coverage by modified coronally advanced flap with and without platelet-rich fibrin: A clinical study. *Indian journal of dental research : official publication of Indian Society for Dental Research.* 2018;29(5):600-604.

29. Francetti L, Weinstein R, Taschieri S, Corbella S. Coronally Advanced Flap With or Without Subepithelial Connective Tissue Graft for the Treatment of Single Recession: 5-Year Outcomes from a Comparative Study. *International journal of periodontics & restorative dentistry.* 2018;38(6):819–825.

30. Rasperini G, Acunzo R, Pellegrini G, et al. Predictor factors for long-term outcomes stability of coronally advanced flap with or without connective tissue graft in the treatment of single maxillary gingival recessions: 9 years results of a randomized controlled clinical trial. *Journal of clinical periodontology.* 2018;45(9):1107‐1117.

31. Rasperini G, Codari M, Paroni L, et al. The Influence of Gingival Phenotype on the Outcomes of Coronally Advanced Flap: A Prospective Multicenter Study. *Int J Periodontics Restorative Dent.* 2020;40(1):e27-e34.

32. Gil S, de la Rosa M, Mancini E, et al. Coronally advanced flap achieved higher esthetic outcomes without a connective tissue graft for the treatment of single gingival recessions: a 4-year randomized clinical trial. *Clinical oral investigations.* 2021;25(5):2727‐2735-2727‐2735.

33. Evginer MS, Olgun E, Parlak HM, Dolgun AB, Keceli HG. Comparison of two techniques in gingival recession treatment: a randomized one-year clinical follow-up study. *Dental and medical problems.* 2022;59(1):121‐130.

34. Garg A, Singh V, Singh A, Mall N. Modified Coronally Positioned Flap for Isolated Gingival Recession, Evaluated with Root coverage Esthetic Score (RES) System. *Journal of Pharmaceutical Negative Results.* 2022;13:122-128.

35. Lombardo G, Tandurella M, Signoriello A, Tomizioli N, Pighi J. Root coverage at upper incisors: Clinical and aesthetic outcomes. *Journal of Clinical Periodontology.* 2022;49:201.

36. Nath J, Changmai A, Bhattacharjee K, Phukan AH, Chakraborty D, Das U. Management of gingival recession by coronally advanced flap with and without amniotic membrane: a clinical study. *Journal of pharmacy & bioallied sciences.* 2022;14(5):S486‐S489.

37. Potharaju SP, Prathypaty SK, Chintala RK, et al. Comparative efficacy of coronally advanced flap with and without guided tissue regeneration in the management of gingival recession defects: a split-mouth trial. *Annals of African medicine.* 2022;21(4):415‐420.

38. Sharma A, Wadhawan A. Comparative evaluation of coronally advanced flap with and without Biomesh® membrane for the treatment of localized gingival recession defects - a clinical study. *Journal of medicine and life.* 2022;15(5):705‐716.

39. Cairo F, Cortellini P, Barbato L, et al. Long-term comparison of root coverage procedures at single RT2 maxillary gingival recessions: ten-year extension results from a randomized, controlled clinical trial. *Journal of clinical periodontology.* 2023;50(4):511‐519.

40. Jagtap A, Mangalekar SB, Kamble P. Clinical Evaluation of Coronally Advanced Flap With or Without Advance-Platelet Rich Fibrin Membrane in the Treatment of Miller's Class-II Localized Gingival Recession: A Clinical Study. *Cureus.* 2023;15(2):e34919.

41. Parlak HM, Yilmaz BT, Durmaz MH, Toz H, Keceli HG. The effects of vertically coronally advanced flap and free gingival graft techniques on shallow vestibule: a randomized comparative prospective trial. *Clinical oral investigations.* 2023;27(12):7425‐7436.
